# Knowledge of Polycystic Ovary Syndrome and Its Associated Symptoms Among Women of Reproductive Age Across Selected Healthcare Facilities in Lagos State, Nigeria

**DOI:** 10.7759/cureus.113121

**Published:** 2026-07-21

**Authors:** Lateef A Akinola, Gideon N Inyangudo, Philip A Iduh

**Affiliations:** 1 Obstetrics and Gynaecology, Medison Specialist Women's Hospital, Lagos, NGA; 2 Research, Medison Specialist Women's Hospital, Lagos, NGA; 3 Prevention, Care and Treatment, Institute of Human Virology, Lagos, NGA

**Keywords:** clinical symptoms of pcos, knowledge of pcos, obstetrics and gynaecology clinics, polycystic ovary syndrome, women of reproductive age group

## Abstract

Background

Despite the increase in the global prevalence of polycystic ovary syndrome (PCOS) and its associated health risks, limited awareness and knowledge persist, resulting in delayed diagnosis and management of the condition.

Objectives

The study was designed to assess the knowledge of PCOS and its associated self-reported symptoms among women of reproductive age in Lagos State, Nigeria.

Methods

The study was a prospective cross-sectional study conducted among 321 women aged 18-45 at Lagos State University Teaching Hospital, Ikeja, and Lagos Island Maternity Hospital. Participants were recruited from the obstetrics and gynecology clinics, excluding pregnant women and those previously diagnosed with PCOS. Data on socio-demographic characteristics, knowledge of PCOS, and clinical symptoms were collected using an adapted self-administered questionnaire. Knowledge of PCOS was measured using 10-item questions. Statistical analyses included descriptive statistics and chi-square tests for categorical variables. The significance level was set at p-values less than 0.05.

Results

The mean BMI of women was 25.72 ± 4.80, with more than half (166; 51.7%) being either overweight or obese. The major self-reported clinical symptoms of PCOS include acne (129, 40.2%), subfertility (149, 46.4%), and irregular menstrual cycles (88, 27.4%). Less than half (130; 40.5%) of the respondents were aware of PCOS, and less than one-third (83; 25.9%) had adequate knowledge of the condition. Respondents’ occupation (p = 0.0001), level of education (p = 0.0001), religion (p = 0.003), and parity (p = 0.0001) were significantly associated with knowledge of PCOS.

Conclusion

This study's findings highlight the need to strengthen health education efforts aimed at improving the awareness and understanding of PCOS among women.

## Introduction

Polycystic ovary syndrome (PCOS), currently known as polyendocrine metabolic ovarian syndrome (PMOS), is a major endocrine and metabolic condition affecting women and often becomes evident during their reproductive years [1]. Although the exact cause is unknown, its etiology has been linked to several genetic, environmental, and lifestyle factors [2].

Globally, it affects approximately 2-20% of women, with up to 70% of cases remaining undiagnosed [1,3]. Studies in Nigeria have reported a prevalence ranging from 6.2% to 27.6% [4].

Due to its complex presenting symptoms, PCOS is regarded as a common metabolic and endocrine condition [5,6]. As a metabolic disorder, it is associated with obesity, insulin resistance, dyslipidemia, and a higher risk of type 2 diabetes [1,7]. As an endocrine disorder, it is presented with decreased levels of follicle-stimulating hormone (FSH), increased levels of luteinising hormone (LH), and a high level of serum insulin, which stimulates excess secretion of testosterone, resulting in hyperandrogenism [8,9]. Consequently, the clinical features of hyperandrogenism include hirsutism, alopecia, and severe acne [10].

Furthermore, its associated hormonal imbalance causes ovulatory dysfunctions, resulting in the development of multiple undeveloped follicles in the ovaries [7,11]. Additionally, women with this condition often experience psychological disabilities such as anxiety, low self-esteem, and depression due to physical changes and comorbidities [12,13].

According to the Rotterdam criteria, PCOS is diagnosed when at least two of the following criteria are present: clinical or biochemical signs of hyperandrogenism, persistent ovulatory dysfunction, and polycystic ovaries following ultrasonography [14]. However, while PCOS may start as early as puberty, it is mostly diagnosed in the reproductive ages, especially when women are faced with infertility issues [1]. Delay in its diagnosis and management can result in long-term reproductive, metabolic, and psychological health complications [15].

Despite the health risks of PCOS and its rising incidence, limited knowledge and awareness, which may result in delayed diagnosis and subsequent management, persist [16-18]. In Nigeria, studies have been characterized by low knowledge among women [17,19], with a few studies conducted among women in the southwest region of Nigeria. Hence, the primary objective of this study was to assess the knowledge level and awareness of PCOS among women of reproductive age in Lagos State, Nigeria. The secondary objectives were to assess the prevalence of self-reported symptoms and identify factors associated with knowledge of the condition among the study population. Findings from this study may aid in identifying specific areas for intervention to improve PCOS knowledge and, consequently, enhance early detection and management.

## Materials and methods

Study design and population

This was a prospective study conducted among 321 non-pregnant women aged 18-45 years attending the gynecology clinics at the Lagos State University Teaching Hospital (LASUTH) and Lagos Island Maternity Hospital (LIMH).

Eligibility criteria

Non-pregnant women aged 18-45 who had not been previously diagnosed with PCOS were recruited for this study. Participants were recruited at the clinics irrespective of their presenting complaints.

Sampling technique and sample size calculation

The sample size was calculated using Leslie Fischer's formula for the calculation of sample size in populations of more than 10,000 ($n = \frac{Z^2pq}{d^2}$). Where n = estimated minimum sample size; z = level of significance at a 95% confidence level (1.96); p = 40.43%, being the proportion of women who were aware of PCOS in a previous study conducted by Ibiye et al. in Nigeria [19]; d was set at a level of precision of 0.05%; and q = 1 - p = 0.6. The estimated sample size for this study was 370.

Sampling techniques

Participants in this study were selected using a two-stage sampling technique. In stage one, two health facilities (one tertiary and one secondary), providing obstetrics and gynecological services, were purposively selected. Obstetric and gynecology clinics at Lagos State University Teaching Hospital (LASUTH) and Lagos Island Maternity Hospital (LIMH) are a few of the main such public clinics in Lagos State. In the second stage, consenting women attending clinic days at both hospitals were recruited consecutively until the predetermined sample size was achieved.

Data collection

Two research assistants were trained on the study protocol, including the data collection tool and ethical compliance. After obtaining informed consent from participants, data were collected using a semi-structured questionnaire (Appendices). All questions were written in English. The questionnaire, which was developed after reviewing relevant literature, was divided into three sections. Section A contains questions on the socio-demographic and obstetric characteristics of the respondents; Section B contains questions assessing the clinical symptoms of PCOS; and Section C contains questions assessing the PCOS knowledge of the respondents. All clinical symptoms experienced by participants were self-reported and not established through any form of clinical and laboratory evaluation.

To ensure basic content validity of the tool, the initial version of the questionnaire was reviewed by a consultant obstetrician and gynecologist. Following the review, the tool was pre-tested among women at two (2) secondary health facilities not included in the study. Findings from the pre-testing exercise were used to further modify the questionnaire.

Operational definition of terms

Sub-infertility: This condition was defined as participants reporting experiencing difficulty conceiving after at least 12 months of regular, unprotected sexual intercourse.

Menstrual irregularity: This condition was defined as participants reporting experiencing short menstrual cycles of less than 21 days, cycles longer than 35 days, or fewer than 8 per year.

Alopecia: This condition was defined as participants reporting experiencing scalp hair loss or thinning.

Acne: The condition was defined as participants reporting the presence of pimples or acne on their face, back, or chest. 

Hirsutism: This condition was defined as participants reporting experiencing the growth of excessive coarse hair in areas where women naturally have little or no hair, such as the face, back, chest, or abdomen.

Measurement of variables

The main outcome variable in this study was the knowledge of respondents about PCOS. The independent variables included socio-demographics and obstetric characteristics of the respondents. Respondent knowledge was assessed using a ten (10)-item questionnaire. Each correct response was scored a maximum of one (1), while an incorrect response was scored zero (0). The knowledge scaled score was computed by summing the 10 knowledge variables, with 0 as the lowest possible score and 10 as the highest possible score. Consistent with previous studies [20,21], respondents with ≥ 50% of the expected composite knowledge score were categorized as having good knowledge, while those with a composite score < 50% were classified as having poor knowledge.

Data analysis

The data were analyzed using the Statistical Package for Social Sciences (SPSS) version 25.0 (IBM Corp., Armonk, NY). The categorical data were presented using sample frequencies and percentages. The continuous data were reported using descriptive statistics such as the mean. Associations between the dependent variables and independent variables were analyzed using the chi-square test, and p-values less than 0.05% were regarded as significant.

Ethical approval

Ethical approval to conduct the study was obtained from LASUTH with protocol number LREC/06/10/1853. Informed consent was obtained from the respondents before the questionnaires were administered. The confidentiality of all respondents was guaranteed by excluding the use of identifiers such as names, addresses, and other information that could reveal the identity of the research participants.

## Results

Socio-demographic and obstetric characteristics of study participants

Of the 370 women approached, 321 gave consent and completed the questionnaire. The average age of participants was 28.66 years (SD = 4.8), while the majority of the respondents were aged 20-29 years (179; 58.8%) and 30-39 years (132; 41.1%). More than one-third (132; 41.1%) of the respondents were involved in skilled work, and most (170; 53.0%) were married. The majority (224; 69.8%) had tertiary education, and 195 (60.7%) were Christians. The mean BMI was 25.72 ± 4.8 kg/m², with more than half (166; 51.7%) being either overweight or obese. More than half (173; 53.9%) had never been pregnant (Table 1).

**Table 1:** Socio-demographic and obstetric characteristics of study participants (n = 321) BMI: body mass index

Variables	Categories	Value
Age	Less than 20 years	5 (1.6%)
	20 - 29 years	179 (55.8%)
	30 - 39 years	132 (41.1%)
	40 years and above	5 (1.6%)
Mean ± SD		28.66 ± 4.80 (years)
Occupation	Unemployed	38 (11.8%)
	Unskilled labor	64 (19.9%)
	Skilled labor	132 (41.1%)
	Professional	87 (27.1%)
Marital status	Single	147 (45.8%)
	Married	170 (53.0%)
	Divorced	4 (1.2%)
Educational status	No formal education	3 (0.9%)
	Primary	3 (0.9%)
	Secondary	91 (28.3%)
	Tertiary	224 (69.8%)
Religion	Christianity	195 (60.7%)
	Islam	126 (39.3%)
BMI	Underweight	9 (2.8%)
	Normal weight	146 (45.5%)
	Overweight	117 (36.4%)
	Obesity	49 (15.3%)
Mean ± SD		25.72 ± 4.80 (kg/m²)
Parity	Nulliparous	173 (53.9%)
	Multiparous	148 (46.1%)

Symptoms of PCOS among respondents

Furthermore, this study evaluates the common self-reported symptoms of PCOS among women. These include acne, alopecia, hirsutism, irregular menstrual cycles, and subfertility. The main observed clinical symptoms of PCOS include acne (129; 40.2%), subfertility (149; 46.4%), and irregular menstrual cycles (88; 27.4%) (Table 2).

**Table 2:** Symptoms of PCOS among respondents (n = 321) PCOS: polycystic ovary syndrome

Variables	Frequency (%)
Acne	
Present	129 (40.2)
Absent	192 (59.8)
Alopecia	
Present	10 (3.1)
Absent	311 (96.9)
Hirsutism	
Present	7 (2.2)
Absent	314 (97.8)
Menstrual pattern	
Irregular	88 (27.4)
Regular	233 (72.6)
Sub-fertility	
Yes	149 (46.4)
No	90 (28.0)
Not applicable	82 (25.5)

Knowledge of PCOS in the study population

**Figure 1:**
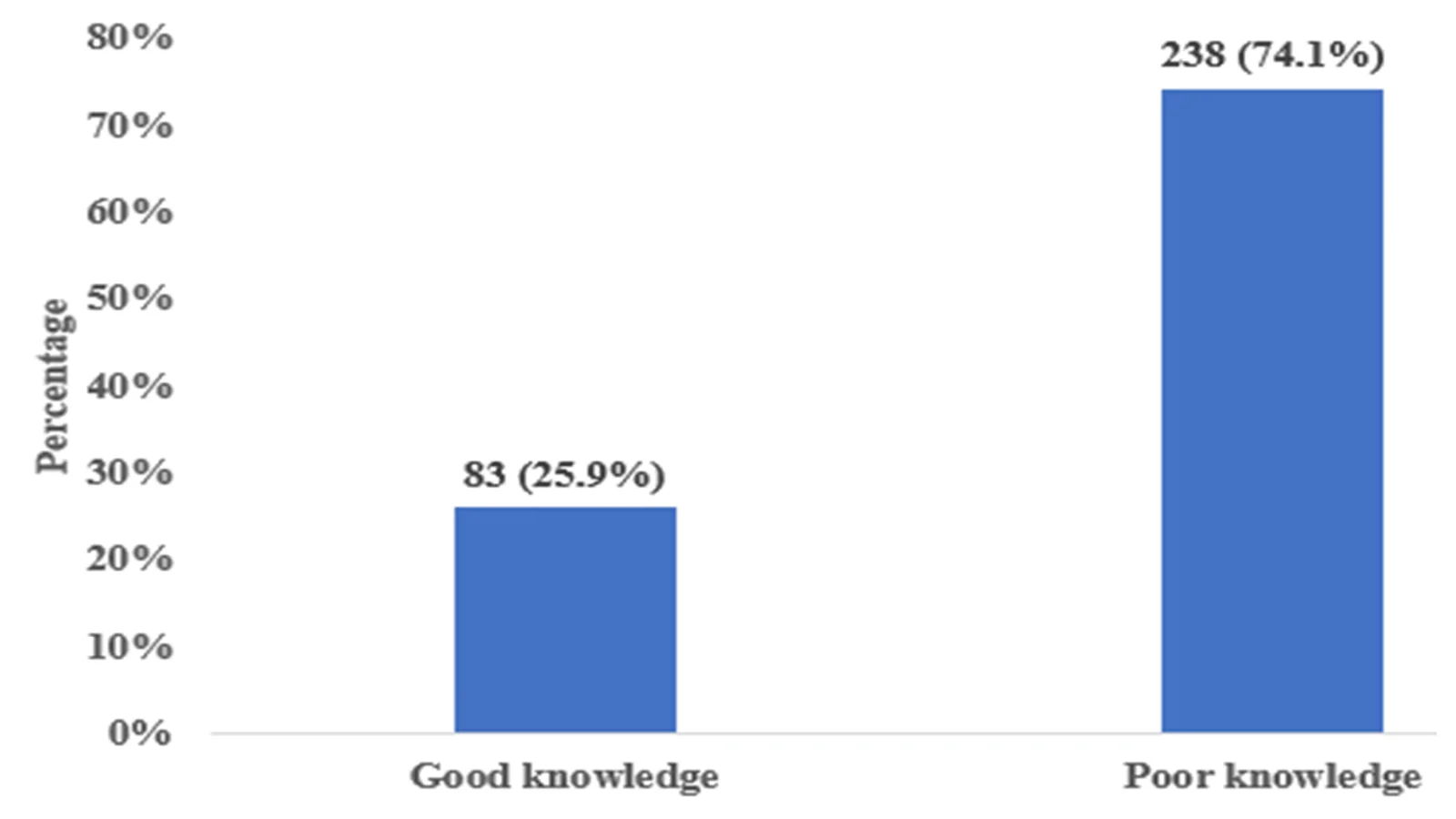
Knowledge score of respondents about PCOS (n = 321) PCOS: polycystic ovary syndrome

A detailed breakdown of respondents’ responses to knowledge questions is shown in Table 3. Generally, participants in this study had low knowledge of PCOS. When asked if they had heard about the condition, only 130 (40.5%) had heard of PCOS prior to participation. For those who had heard, their main sources of information were healthcare workers (67, 51.5%) and the internet (49, 37.7%). Regarding what they know about PCOS, most (88; 27.4%) noted it to be associated with irregular menstruation, 78 (24.3%) indicated that it is associated with infertility, 63 (19.6%) indicated that it is associated with the presence of hair in abnormal areas in females, and only 51 (15.9%) agreed that PCOS is associated with the presence of multiple undeveloped follicles in the ovaries. Furthermore, 54 (16.8) of the women revealed that the condition is curable, 76 (23.7) noted that it is a major cause of infertility, 21 (6.5) indicated that it is a chronic/life-long disease, and more than one-quarter (99; 30.8%) identified hormonal imbalance as its key feature. Less than one-quarter (76; 23.7%) of the women mentioned the ovary as the organ mainly affected in PCOS (Table 3). Furthermore, respondents’ knowledge score about PCOS showed that only 83 (25.9%) of the women had good knowledge of PCOS (Figure 1).

**Table 3:** Knowledge of PCOS among respondents (n = 321) PCOS: polycystic ovary syndrome

Knowledge items	Categories	Frequency (%)
Have you heard of polycystic PCOS (polycystic ovarian syndrome)?	Yes	130 (40.5)
	No	191 (59.5)
Sources of information	Health worker	67 (51.5)
	Internet	49 (37.7)
	Friend	8 (6.2)
	Relatives	6 (4.6)
Which of these is associated with PCOS?		
Irregular menstruation	Yes	88 (27.4)
	No	233 (72.6)
Difficulty in getting pregnant	Yes	78 (24.3)
	No	243 (75.7)
Presence of hairs on abnormal areas in females	Yes	63 (19.6)
	No	258 (80.4)
Presence of undeveloped follicles in the ovaries	Yes	51 (15.9)
	No	270 (84.1)
PCOS is curable	Yes	54 (16.8)
	No	267 (83.2)
PCOS is a major cause of infertility	Yes	76 (23.7)
	No	245 (76.3)
PCOS is a chronic/lifelong disease	Yes	21 (6.5)
	No	300 (93.5)
Hormonal imbalance is a key feature of PCOS	Yes	99 (30.8)
	No	222 (69.2)
Which organ of reproduction is affected by PCOS?	The fallopian tubes	6 (1.9)
	The ovaries	76 (23.7)
	The womb	3 (0.9)
	The cervix	2 (0.6)
	I don’t know	234 (72.9)

Association between the knowledge score of PCOS and characteristics of the respondents

There was a significant association between the knowledge of PCOS and some characteristics of the respondents. Knowledge of PCOS was significantly associated with the respondent's occupation (chi-square = 38.26, P-value = 0.0001), level of education (likelihood ratio = 45.952, P-value = 0.0001), religion (chi-square = 9.138, P-value = 0.003), and parity (chi-square = 6.194, P-value = 0.013). However, there was no significant association between the knowledge of PCOS and age or marital status (Table 4).

**Table 4:** Association between knowledge of PCOS and characteristics of the respondents LR: likelihood ratio; χ² = Pearson chi-square; * significant at p < 0.05 The Pearson chi-square test was used for variables where expected cell frequencies were adequate. A likelihood ratio chi-square test was used for variables where some expected cell counts were small. PCOS: polycystic ovary syndrome

Variables	Categories	Good knowledge Frequency (%)	Poor knowledge Frequency (%)	Chi-square	P-value
Age	Less than 20 years	1 (20.0)	4 (80)	LR = 1.086	0.784
	20 - 29 years	43 (24.0)	136 (76.0)		
	30 - 39 years	38 (28.8)	94 (71.2)		
	40 years and above	1 (20)	4 (80)		
Occupation	Unemployed	9 (23.7)	29 (76.3)	χ^2^ = 38.26	0.0001*
	Unskilled labour	2 (3.1)	62 (96.9)		
	Skilled labour	31 (23.5)	101 (76.5)		
	Professional	41 (47.1)	46 (52.9)		
Marital status	Single	44 (29.9)	103 (70.1)	LR = 3.974	0.137
	Married	37 (21.8)	133 (78.2)		
	Divorced	2 (50.0)	2 (50.0)		
Educational status	No formal education	1 (33.3)	2 (66.7)	LR = 45.952	0.0001*
	Primary	0 (0.0)	3 (100.0)		
	Secondary	3 (3.3)	88 (96.7)		
	Tertiary	79 (35.3)	145 (64.7)		
Religion	Christianity	62 (31.8)	133 (68.2)	χ^2^ = 9.138	0.003*
	Islam	21 (16.7)	105 (83.3)		
Parity	Never being pregnant	48 (32.4)	100 (67.6)	χ^2^ = 6.194	0.013*
	Ever pregnant	35 (20.2)	138 (79.8)		

Although PCOS knowledge was significantly associated with the menstrual patterns of women (chi-square = 14.328, p-value = 0.0001), it was not significant with other symptoms such as acne, alopecia, hirsutism, and subfertility (Table 5).

**Table 5:** Association between knowledge of PCOS and PCOS symptoms of the respondents χ² = Pearson chi-square; * significant at p < 0.05 PCOS: polycystic ovary syndrome

Variables	Categories	Good knowledge frequency (%)	Poor knowledge frequency (%)	Chi-square	p-value
Acne	Present	35 (27.1)	94 (72.9)	^χ2^ = 0.183	0.669
	Absent	48 (25.0)	144 (75.0)		
Alopecia	Present	2 (20.0)	8 (80.0)	χ^2^ = 0.185	0.667
	Absent	81 (26.0)	230 (74.0)		
Hirsutism	Absent	79 (25.2)	235 (74.8)	χ^2^ = 3.654	0.056
	Present	4 (57.1)	3 (42.9)		
Menstrual pattern	Irregular	36 (40.9)	52 (59.1)	χ^2^ = 14.328	0.0001*
	Regular	47 (20.2)	186 (79.8)		
Infertility	Yes	38 (25.5)	111 (74.5)	χ^2^ = 0.309	0.858
	No	22 (24.4)	68 (75.6)		
	Not applicable	23 (28.0)	59 (72.0)		

## Discussion

PCOS, although a disease of concern that impacts both the reproductive and social lives of women of reproductive age, is poorly understood in most cases. This study was, however, conducted to assess self-reported symptoms and knowledge of PCOS among women of reproductive age in Lagos State.

A substantial proportion of self-reported symptoms of PCOS observed in this study may suggest a high burden of symptoms suggestive of PCOS among the study population. Thus, there is a need to intensify efforts on awareness education on PCOS and its diagnosis to enhance early detection. Although acne was the most commonly self-reported symptom of hyperandrogenism, the report of other clinical features of hyperandrogenism, such as alopecia (3.1%) and hirsutism (2.2%), was relatively low. These findings may be attributed to several factors, including phenotypic differences in PCOS symptoms across geographical areas, with women in certain locations having a low incidence of hirsutism despite high biochemical hyperandrogenism [22]. Additionally, individual perception of what constitutes alopecia or hirsutism may have influenced the report. Women may normalize mild hair changes or could attribute them to aging, psychosocial stress, or hair-care habits rather than underlying endocrine dysfunction.

Furthermore, the subfertility reported by the majority (46.4%) of the study participants may signify a high reproductive health burden among the study population. However, the 25.5% of participants stating that subfertility was “not applicable” could be explained by variation in reproductive goals and life stages, which need to be taken into account when interpreting fertility-related symptoms.

Despite a substantial proportion of the women reporting symptoms suggestive of PCOS, the level of awareness and knowledge of the condition was found to be low, with less than half of the participants admitting to having ever heard about the condition prior to their participation in the study. This finding underlines a critical gap in reproductive health literacy and the potential risk of late diagnosis and management of the condition among women [23]. Although higher than the 25% previously reported in Anambra State [16], the level of PCOS awareness in this study was lower than the 65% reported among undergraduates in Port Harcourt [24] and the 89% reported in Jordan [25]. Given the rising prevalence of PCOS and its associated metabolic, reproductive, and psychological complications, this finding, which agrees with the 40.43% and 41% awareness levels reported in Port Harcourt [19] and India [26], respectively, is of public health importance. Hence, educational interventions promoting PCOS awareness and other reproductive health issues are essential to enhance women’s ability to make timely and informed reproductive health decisions. Awareness of PCOS in this study did not reflect adequate knowledge, as less than one-third (25.9%) of the women had good knowledge of PCOS compared to the 40.5% awareness level. This observed gap shows that exposure to only basic information about PCOS often may not result in proper understanding of symptoms, causes, and health impact of the condition. Hence, a more comprehensive and intensive health education intervention needs to be implemented.

Healthcare professionals (51.5%), followed by the internet (37.7%), were identified as the common sources of information about PCOS. Conversely, teachers and acquaintances (friends and family) were reported from previous studies conducted in Nigeria [16] and the UAE [27], respectively, as primary sources of information. The disparity in sources of information can be explained partly by the differences in population characteristics of the present study and the aforementioned studies. The fact that healthcare professionals were the main source of information about PCOS in this study signifies their relevance in disseminating reproductive health information and shaping women’s understanding of gynecological conditions such as PCOS. Furthermore, the existence of a possible sociocultural barrier to discussing reproductive health issues like PCOS with family and friends [16] may have explained the low percentage of friends and family as a source of information about PCOS observed in this study. The low knowledge of PCOS observed in this study was in line with previous studies. Low knowledge of PCOS was reported among women in a previous Nigerian study [17]. Similarly, a study conducted in Pakistan found that 63% of the participants gave incorrect information regarding signs and symptoms and complications of PCOS [18]. Conversely, Jaber et al. and Srour et al. reported that the levels of knowledge of PCOS were high among women in Jordan and Lebanon, respectively [25,28]. This disparity may be due to differences in the study location, as the two studies were conducted outside Nigeria. 

The observed overall low knowledge level, despite healthcare workers being the main source of information about PCOS, suggests insufficient integration of PCOS-related education into routine maternal health services, resulting in missed opportunities for patient education during hospital visits. Hence, strengthening health professionals' capacity to deliver structured and comprehensible education on PCOS could significantly improve patients' understanding.

Occupation, education level, religion, parity, and menstrual pattern of participants were significantly associated with knowledge of PCOS in this study. This positive association between educational level and knowledge in this study was consistent with findings from previous studies [25,29], reflecting the role of education in the acquisition of health information and, consequently, improving health literacy. Women with higher levels of education are more likely to be exposed to reproductive health information while attending health-related and scientific events [30]. Also, they can effectively access the internet to acquire health information, thereby gaining more knowledge compared to women with lower levels of education. Furthermore, the knowledge gap across educational levels may be because PCOS is often described using formal medical terminologies that may not be easily understood by people with lower levels of education. Hence, translation of PCOS terminologies and educational materials into relevant local languages can improve understanding of PCOS, thereby impacting the overall health-seeking behaviour of women. The association between parity, menstruation pattern, and knowledge observed in this study highlights the role of reproductive health experiences in shaping women’s knowledge of gynecological disorders. Given that healthcare workers were the main source of information about PCOS, women experiencing any form of menstrual disorders may have acquired adequate knowledge regarding the condition during clinical encounters. This highlights the need for healthcare professionals to incorporate effective patient education about reproductive issues such as PCOS when interacting with patients during their clinical visits.

Limitations

This study may not be generalized to the entire population of women since it only involved women visiting gynecology clinics. The self-reporting of PCOS symptoms may have resulted in misclassification due to recall bias. Also, reliability testing of the study instrument using Cronbach’s alpha was not conducted and is recommended in future studies. Future research involving community-based designs and clinical evaluation of PCOS symptoms is recommended. This can provide more information on the prevalence of PCOS symptoms and a generalized understanding of PCOS knowledge among women.

## Conclusions

Despite a substantial number of the study participants reporting experiencing symptoms suggestive of PCOS, poor knowledge of PCOS was reported. This highlights a possible high risk of delayed diagnosis and management of gynecological disorders, such as PCOS, among the study population. This study's findings, therefore, underscore the need to strengthen health education initiatives aimed at improving the awareness and understanding of PCOS among women. Such initiatives should include strengthening health providers' capacity to deliver structured and patient-centered reproductive health education, designing and deploying educational digital platforms on PCOS to increase awareness coverage, and integrating PCOS-focused education into existing reproductive health services. Additionally, using targeted cultural communication strategies, such as using local languages, can further improve understanding, especially in settings involving women with lower educational levels.

## Appendices

**Table 6:** Questionnaire

SECTION A: PERSONAL INFORMATION		
A1	Age	
A2	Occupation	A. Unemployed B. Unskilled Labor C. Skilled labor D. Professional
A3	Marital status	A. Single B. Married C. Divorced D. Widowed
A4	Ethnicity	A. Yoruba B. Hausa C. Igbo D. Others
A5	Educational status	A. No formal education B. Primary C. Secondary D. Tertiary
A6	Religion	A. Christianity B. Islam C. Traditional
A7	Number of children	-------------------------------
A8	Height	-------------------------------
A9	Weight	-------------------------------
A10	BMI	-------------------------------
SECTION B: SIGNS AND SYMPTOMS OF POLYCYSTIC OVARY SYNDROME (PCOS)		
B1	Have you been pregnant before?	A. Yes B. No
B2	Do you have difficulties getting pregnant?	A. Yes B. No C. Not applicable
B3	How many times have you had your period in the last one year?	______________________
B4	Do you experience acne?	A. Present B. Absent
B5	Do you experience loss of hair (alopecia)?	A. Present B. Absent
B6	Ferriman-Gallwey score:	A. Facial B. Upper limb C. Lower limb D. Chest region E. Back F. Abdomen
SECTION C: KNOWLEDGE OF PCOS		
C1	Have you heard of Polycystic Ovary Syndrome (PCOS)	A. Yes B. No
C2	If yes, where did you hear about it? (Please tick as apply)	A. Friend B. Health worker C. Internet D. Relatives E. Radio F. TV G. Others----------
C3	Which of these is associated with PCOS? (Please tick as apply.)	A. I don’t know. B. Irregular menstruation C. Inability to become pregnant. D. Presence of hairs on abnormal areas in females. E. Presence of undeveloped follicles in the ovary
C4	Is PCOS curable?	A. Yes B. No
C5	Is PCOS a major cause of infertility?	A. Yes B. No
C6	Is PCOS a chronic or lifelong disease?	A. Yes B. No
C7	Is hormonal imbalance a key feature of PCOS?	A. Yes B. No
C8	Which of these organs of reproduction is affected by PCOS?	A. The fallopian tubes B. The cervix C. The ovaries D. The womb E. I don’t know

## References

[1] (2026). WHO. Polycystic ovary syndrome. https://www.who.int/news-room/fact-sheets/detail/polycystic-ovary-syndrome.

[2] Singh S, Pal N, Shubham S, Sarma DK, Verma V, Marotta F, Kumar M (2023). Polycystic ovary syndrome: etiology, current management, and future therapeutics. J Clin Med.

[3] Deswal R, Narwal V, Dang A, Pundir CS (2020). The prevalence of polycystic ovary syndrome. A brief systematic review. J Hum Reprod Sci.

[4] Akpata CB, Uadia PO, Okonofua FE (2018). Prevalence of polycystic ovary syndrome in Nigerian women with infertility: a prospective study of the three assessment criteria. Open J Obstet Gynecol.

[5] Siam SG, Soliman B.S, Ali MR, Abdallah OA (2020). Prevalence of polycystic ovarian syndrome in young adult unmarried females attending Zagazig University Hospital outpatient clinic. Egypt J Hosp Med.

[6] Pasquali R (2018). Contemporary approaches to the management of polycystic ovary syndrome. Ther Adv Endocrinol Metab.

[7] Ajmal N, Khan SZ, Shaikh R (2019). Polycystic ovary syndrome (PCOS) and genetic predisposition: a review article. Eur J Obstet Gynecol Reprod Biol X.

[8] Faraj RM, Jawad AH, Badr AH (2019). Effect of body mass index on abnormal ovarian secretion hormones among Iraqi women with polycystic ovarian syndrome (PCOS). Al-Nahrain J Sci.

[9] American College of Obstetricians and Gynecologists (ACOG (2022). American College of Obstetricians and Gynecologists (ACOG). Polycystic ovary syndrome (PCOS). https://www.acog.org/womens-health/faqs/polycystic-ovary-syndrome-pcos.

[10] Spritzer PM, Marchesan LB, Santos BR, Fighera TM (2022). Hirsutism, normal androgens and diagnosis of PCOS. Diagnostics (Basel).

[11] Rosenfield RL (2020). Current concepts of polycystic ovary syndrome pathogenesis. Curr Opin Pediatr.

[12] Almeshari WK, Alsubaie AK, Alanazi RI, Almalki YA, Masud N, Mahmoud SH (2021). Depressive and anxiety symptom assessment in adults with polycystic ovarian syndrome. Depress Res Treat.

[13] Çoban ÖG, Tulacı ÖD, Adanır AS, Önder A (2019). Psychiatric disorders, self-esteem, and quality of life in adolescents with polycystic ovary syndrome. J Pediatr Adolesc Gynecol.

[14] The Rotterdam ESHRE/ASRM‐sponsored PCOS consensus workshop group (2004). Revised 2003 consensus on diagnostic criteria and long-term health risks related to polycystic ovary syndrome (PCOS). Hum Reprod.

[15] Shukla A, Rasquin LI, Anastasopoulou C (2025). Polycystic ovarian syndrome. StatPearls [Internet].

[16] Ejiofor DU, Chinwe UE (2023). Awareness of polycystic ovary syndrome through Facebook: study of adolescent secondary school girls in Anambra State. African Scholars Multidisciplinary Journal (ASMJ).

[17] Omagbemi A, Ezinne C, Ofeoritse A (2020). Current knowledge and perceptions of women about polycystic ovarian syndrome in Nigeria. Int J Sci Healthc Res.

[18] Hameed L, Khan MN, Farooq AD, Siddiqui SM (2024). Knowledge and prevalence of polycystic ovarian syndrome among undergraduate female students in Karachi. J Popul Ther Clin Pharmacol.

[19] Ibiye OE, Ogbu OS, Zigabelbari ZV (2021). Assessment on the level of knowledge of women of reproductive age group about polycystic ovarian syndrome (PCOS) and its effect on reproductive hormones. Sci Res J.

[20] Goh JE, Farrukh MJ, Keshavarzi F (2022). Assessment of prevalence, knowledge of polycystic ovary syndrome and health-related practices among women in Klang Valley: a cross-sectional survey. Front Endocrinol (Lausanne).

[21] Mohammad A.U and Jiya F.B (2024). Nutritional knowledge of in-school adolescents in Sokoto, North-Western Nigeria. Int J Sci Rep.

[22] Kemfang JDN, Omgba JYO, Ngo EU, Chebu CAN, Fouogue JT, Kingsley O, Eugène S (2022). Prevalence and phenotypic features of polycystic ovary syndrome among patients attending gynaecology clinic in two referral hospitals in Yaoundé, Cameroon. Int J Reprod Contracept Obstet Gynecol.

[23] Nidhi R, Padmalatha V, Nagarathna R, Amritanshu R (2011). Prevalence of polycystic ovarian syndrome in Indian adolescents. J Pediatr Adolesc Gynecol.

[24] Olotu J, Okon M (2020). Awareness of polycystic ovarian syndrome among young female adults in Nigeria. EC Endocrinol Metab Res.

[25] Jaber RM, Aripin A, Allias N, Omar S, Kamal NR, Dwekat O (2022). Knowledge and attitudes towards polycystic ovary syndrome. Afr J Reprod Health.

[26] Patel J, Rai S (2018). Polycystic ovarian syndrome (PCOS) awareness among young women of central India. Int J Reprod Contracept Obstet Gynecol.

[27] Zaitoun B, Al Kubaisi A, AlQattan N, Alassouli Y, Mohammad A, Alameeri H, Mohammed G (2023). Polycystic ovarian syndrome awareness among females in the UAE: a cross-sectional study. BMC Womens Health.

[28] Srour I, Salhab S, Skaiki H, Sakr S, Sheet I (2024). Assessment of prevalence, knowledge of polycystic ovary syndrome and health-related practices among female nurses in Lebanon. Open Public Health J.

[29] Alruwaili GA, Mohammad SM, Almoaibed FM (2020). General public awareness toward polycystic ovarian syndrome among females in Saudi Arabia. Int J Med Dev Ctries.

[30] Anwer Z, Ahmed F, Al-Tukmagi H (2017). Health literacy among women with different educational States in Baghdad. Asian J Pharm Res Health Care.

